# From selected multi-sensory dimensions to positive word of mouth: Data on what really drives generation z consumers to be attached to quick service restaurants in bloemfontein, south africa?

**DOI:** 10.1016/j.dib.2020.106279

**Published:** 2020-09-07

**Authors:** Eugine Tafadzwa Maziriri, Tarisai Fritz Rukuni, Tinashe Chuchu

**Affiliations:** a Department of Business Management, University of the Free State, Bloemfontein, South Africa; b Department of Marketing Management, University of Pretoria, Pretoria, South Africa

**Keywords:** Multi-sensory dimensions, Consumer attitudes, Restaurant patronage intention, Food purchase decision, Food consumption satisfaction, Restaurant attachment, Repurchase intention, Positive word of mouth

## Abstract

This article presents raw inferential statistical data that determined the how selected multi-sensory dimensions such as sight, sound and smell would influence consumer attitudes towards quick-service restaurants, restaurant patronage intention, food purchase decision, food consumption satisfaction, restaurant attachment, repurchase intention and positive word of mouth in South African quick-service restaurants. To test the conceptual model an online questionnaire was used to collect data from Generation Z restaurant consumers within the metropolitan area of Bloemfontein, South Africa. The data were analysed using a quantitative approach. Reliability and validity were confirmed. The data was presented using Structural Equation modeling (SEM) using the Smart PLS program. The analysis of the SEM path shows estimates of the interconnectivity of the major constructs in the data. The findings from this dataset show that sight, sound and smell had on consumer attitudes towards quick-service restaurants and restaurant patronage intention. In addition, consumer attitudes towards quick-service restaurants and restaurant patronage intention had a positive influence on food purchase decisions. Food purchase decisions positively and significantly influenced food consumption satisfaction. Additionally, food consumption satisfaction positively and significantly influenced restaurant attachment, repurchase intention and positive word of mouth. Furthermore, restaurant attachment had a positive influence on repurchase intention and repurchase intention had a positive influence on positive word of mouth. Moreover, surprisingly, restaurant attachment had a negative and an insignificant influence on positive word of mouth.

## Specifications Table

**Table:** 

Subject	Business and Marketing
Specific subject area	Consumer behaviour, retailing, restaurant consumption behaviour
Type of data	Tables and figures
How data were acquired	Data was gathered significantly through the dissemination of online questionnaires to Generation Z consumers within the Bloemfontein Metropolitan region
Data format	Raw, analysed, descriptive and statistical data
Parameters for data collection	To qualify for inclusion in the sample the participants had to be Generation Z restaurant consumers within the Bloemfontein metropolitan area.
Description of data collection	An online questionnaire was used to collect data from 381 Generation Z restaurant consumers within the metropolitan area of Bloemfontein. The questionnaire is provided as a supplementary file.
Data source location	University of the Free State, Bloemfontein, South Africa.
Data accessibility	Data is included in this article

## Value of the Data

• The data helps explain how multi-sensory dimensions such as sight, sound and smell would influence consumer attitudes towards quick-service restaurants, restaurant patronage intention, food purchase decision, food consumption satisfaction, restaurant attachment, repurchase intention and positive word of mouth in South African and African quick-service restaurants as a whole.
• The data can be used to enlighten restaurant and marketing managers on the importance of multi-sensory dimensions, as well as how they can be beneficial to enhancement of consumer attitudes towards and consumer behavioural intentions.
• The data can be used as a springboard for further discourse on how restaurant and marketing managers could enhance positive word of mouth in quick-service restaurants.
• Data presented in this data article provides retail strategies which might be utilised to win market share.
• The data does not involve any control variables but further research could consider using any one of the constructs of this study as control variables.

## 1 Data Description

**Fig. 1:**
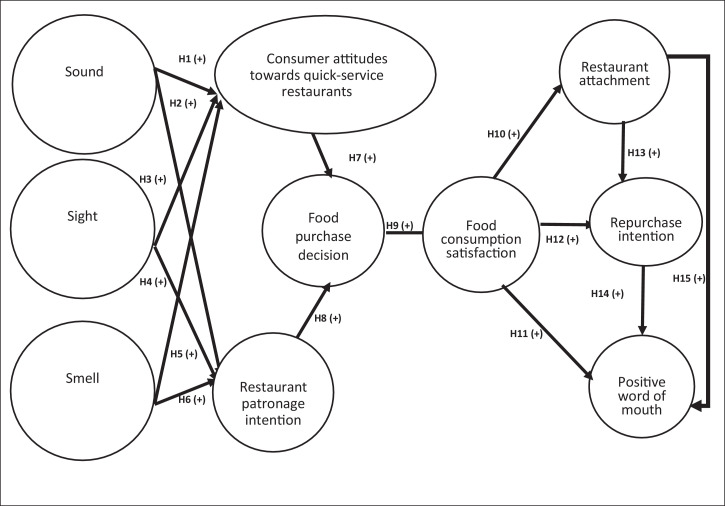
Maziriri sensory trigger model.

**Fig. 2:**
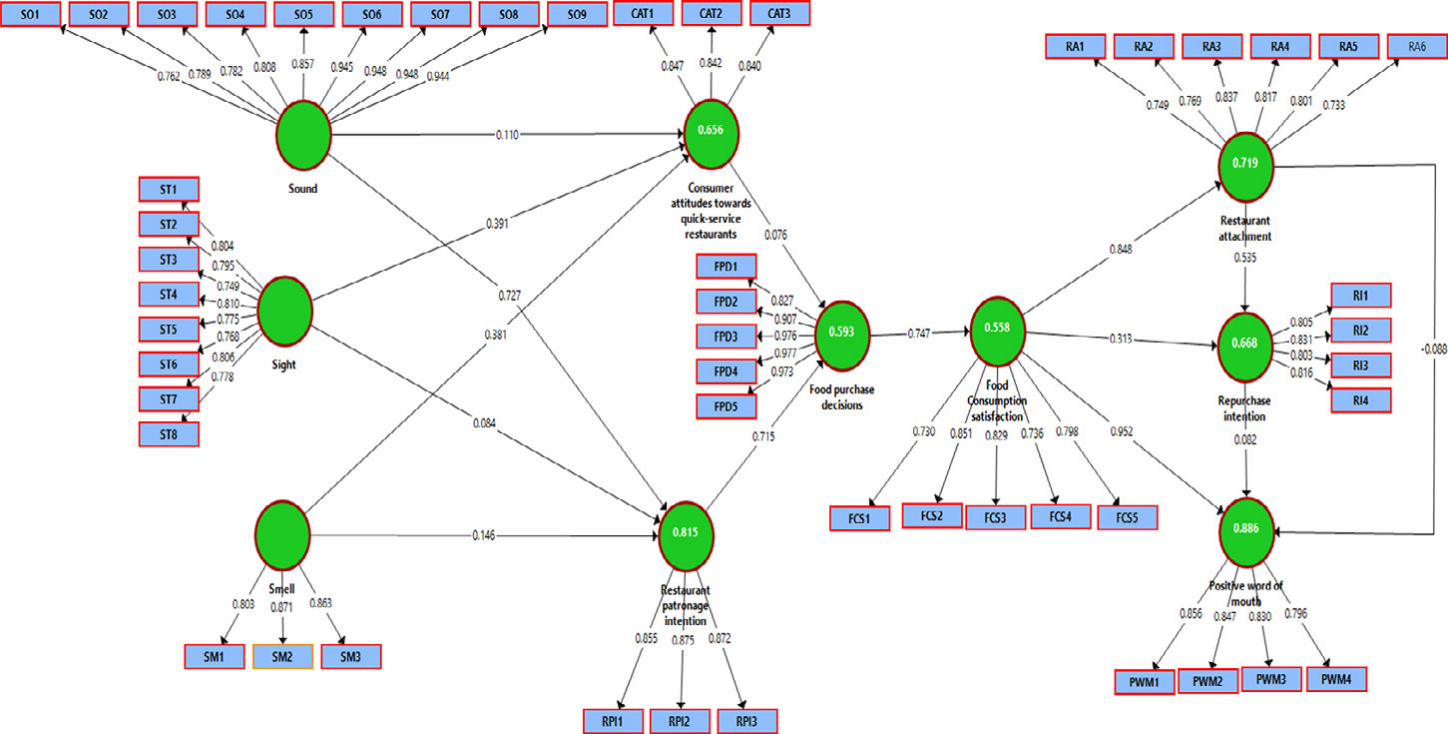
Structural model.

Raw data was collected on generation Z consumers’ behaviour regarding quick-service restaurants. The data files comprise of two supplementary files, namely the dataset in Excel (file 1) and the questionnaire in MS Word (file 2). The processed data is then presented through four tables and two figures. First, the researchers, drafted a conceptual model (Fig 1) which served as a guide to test the data in a statistical manner. Table 1 presents the sample profile showing demographic data of the participants. Measurement accuracy assessment data is described in table 2, presenting the Cronbach's alpha value, composite reliability, average variance extracted (AVE) and factor loadings. Fig 2 describes the structural model which depicts the research constructs post-analysis. Table 3 provides the model fit summary while table 4 depicts the outcomes of structural equation model analysis where proposed hypotheses, path coefficients (β) and p-values are presented.

**Table 1:** Sample profile.

Characteristics	Frequency	%
Age		
18 years old	28	7,4
19 years old	32	8,4
20 years old	14	3,7
21 years old	193	50,8
22 years old	43	11,3
23 years old	40	10,5
24 years old	21	5,5
25 years old	9	2,4
Total	380	100
Gender		
Male	212	55,8
Female	161	42,4
Prefer not to say	7	1,8
Total	380	100
Year of study		
1st year	93	24,5
2nd year	110	28,9
3rd year	85	22,4
Post graduate study	92	24,2
Total	380	100
Allowance usually received per month		
Less than R500	35	9,2
R501 – R1000	90	23,7
R1001-R1500	46	12,1
R1501-R2000	135	35,5
More than R2000	74	19,5
Total	380	100
How often do you eat from quick-service restaurants		
Everyday	2	0,5
A few times a week	61	16,1
A few times a month	116	30,5
Once in a while	201	52,9
Total	380	100

**Table 2:** Measurement accuracy assessment.

Research	PLS	Scale item		Cronbach's	Composite	Average variance	Factor
constructs	code item	Mean	SD	alpha value	reliability	extracted (AVE)	loadings
Sound	SO1	3.868	0.777	0.958	0.965	0.754	0.762
	SO2	3.958	0.717				0.789
	SO3	3.974	0.757				0.782
	SO4	3.932	0.729				0.808
	SO5	3.871	0.796				0.857
	SO6	3.892	0.730				0.945
	SO7	3.892	0.727				0.948
	SO8	3.887	0.722				0.948
	SO9	3.895	0.721				0.944
Sight	ST1	4.074	0.757	0.912	0.928	0.618	0.804
	ST2	4.026	0.684				0.795
	ST3	3.989	0.736				0.749
	ST4	4.034	0.726				0.810
	ST5	3.963	0.746				0.775
	ST6	3.866	0.798				0.768
	ST7	3.982	0.720				0.806
	ST8	4.037	0.717				0.778
Smell	SM1	4.047	0.702	0.801	0.883	0.716	0.803
	SM2	3.892	0.762				0.871
	SM3	3.868	0.784				0.863
Consumer attitudes	CTA1	4.000	0.740	0.797	0.880	0.710	0.847
	CTA2	4.000	0.764				0.842
	CTA3	4.082	0.715				0.840
Food Purchase decision	FPD1	3.937	0.730	0.962	0.971	0.872	0.827
	FPD2	3.874	0.791				0.907
	FPD3	3.895	0.732				0.976
	FPD4	3.889	0.728				0.977
	FPD5	3.897	0.724				0.973
Restaurant patronage intention	RPI1	3.868	0.777	0.835	0.901	0.752	0.855
	RPI2	3.961	0.719				0.875
	RPI3	3.974	0.757				0.872
Food Consumption satisfaction	FCS1	3.897	0.720	0.849	0.892	0.625	0.730
	FCS2	4.079	0.757				0.851
	FCS3	4.032	0.684				0.829
	FCS4	3.995	0.736				0.736
	FCS5	4.029	0.723				0.798
Restaurant attachment	RA1	3.963	0.750	0.875	0.906	0.616	0.749
	RA2	3.868	0.800				0.769
	RA3	3.987	0.720				0.837
	RA4	4.034	0.716				0.817
	RA5	4.050	0.703				0.801
	RA6	3.895	0.764				0.733
Repurchase intention	RI1	3.871	0.786	0.830	0.887	0.662	0.805
	RI2	3.995	0.740				0.831
	RI3	4.003	0.766				0.803
	RI4	4.092	0.714				0.816
Positive word of mouth	PWM1	3.895	0.725	0.853	0.900	0.693	0.856
	PWM2	3.897	0.724				0.847
	PWM3	4.076	0.759				0.830
	PWM4	4.045	0.689				0.796

**Table 3:** Model fit summary.

Estimated Model	
SRMR	0.070
d_ULS	1.727
d_G1	0.941
d_G2	0.783
Chi-Square	1919.097
NFI	0.851

**Table 4:** Outcomes of structural equation model analysis.

Path	Hypothesis	Path coefficients (β)	T- Statistics	P-value	Decision
Sound -> Consumer attitudes towards quick-service restaurants	H1(+)	0.110	2.284	0.023	Positive and significant
Sound -> Restaurant patronage intention	H2(+)	0.727	22.212	0.000	Positive and significant
Sight -> Consumer attitudes towards quick-service restaurants	H3(+)	0.391	7.379	0.000	Positive and significant
Sight -> Restaurant patronage intention	H4 (+)	0.084	2.148	0.032	Positive and significant
Smell -> Consumer attitudes towards quick-service restaurants	H5 (+)	0.381	6.824	0.000	Positive and significant
Smell -> Restaurant patronage intention	H6 (+)	0.146	3.526	0.000	Positive and significant
Consumer attitudes towards quick-service restaurants -> Food purchase decisions	H7 (+)	0.076	1.618	0.106	Positive and insignificant
Restaurant patronage intention -> Food purchase decisions	H8 (+)	0.715	15.425	0.000	Positive and significant
Food purchase decisions_ -> Food Consumption satisfaction	H9 (+)	0.747	24.861	0.000	Positive and significant
Food Consumption satisfaction -> Restaurant attachment	H10 (+)	0.848	40.196	0.000	Positive and significant
Food Consumption satisfaction -> Positive word of mouth	H11 (+)	0.952	21.966	0.000	Positive and significant
Food Consumption satisfaction -> Repurchase intention	H12 (+)	0.313	4.687	0.000	Positive and significant
Restaurant attachment -> Repurchase intention	H13(+)	0.535	8.461	0.000	Positive and significant
Repurchase intention -> Positive word of mouth	H14 (+)	0.082	2.304	0.022	Positive and significant
Restaurant attachment -> Positive word of mouth	H15 (+)	−0.088	1.736	0.083	Negative and insignificant

## 2 Experimental Design, Materials and Methods

The data presented was based on a quantitative approach. A descriptive research design was adopted to obtain the opinions of consumers concerning the multi-sensory dimensions, consumer attitudes towards and consumers behavioural intentions. An online survey method was considered an appropriate data collection method because it allows for the collection of standardised data that permits the researcher to produce information for answering the how, who, what and when questions regarding the subject matter. Generation Z student consumers within the Bloemfontein metropolitan area. To test the data, the researchers proposed the model whereby sound, sight and smell were the predictor variables. Consumer attitudes towards quick-service restaurants, restaurant patronage intention, food purchase decision, food consumption satisfaction, were the mediating variables. Moreover, restaurant attachment, repurchase intention and positive word of mouth were the outcome variables. The researchers had to propose a model to test the validity of the proposed model as well as to determine if the data, which has been collected in the field, fits well with the proposed conceptual model.

### 2.1 Assessment of the goodness of fit (GoF)

Overall, R² for consumer attitudes, restaurant patronage intention, food purchase decision, food consumption satisfaction, restaurant attachment, repurchase intention and positive word of mouth in Fig. 2 indicate that the research model explains 65.6%, 81.5%, 59.3%, 55.8%, 71.9%, 66.8% and 88.6% respectively, of the variance in the endogenous variables. The following formulae given by [1], the global GoF statistic for the research model was calculated using the equation:

$$\text{Goodness}\;\text{of}\;\text{Fit}=\;\sqrt[{2}]{\left(\text{average}\;\text{of}\;\text{all}\;\text{AVEs}\;\text{values}*\;\text{average}\;\text{of}\;\text{all}\;R^{2}\right)}$$

$$\sqrt[{2}]{0.701*0.400\;}=\;0.53$$

where AVE represents the average of all AVE values for the research variables while *R²* represents the average of all R² values in the full path model. The calculated global GoF is 0.53, which exceeds the threshold of GoF > 0.36 suggested by [2]. Therefore, it can be concluded that the research model has a good overall fit.

### 2.2 The standardized root mean square residual (SRMR)

The SRMR is an index of the average of standardized residuals between the observed and the hypothesized covariance matrices [3]. The SRMR is a measure of estimated model fit. When SRMR = <0.08, then the study model has a good fit [4], with a lower SRMR being a better fit. Table 3 shows the theoretical model's SRMR was 0.07, which revealed that the model had a good fit, whereas the Chi-Square was equal to 1919.097 and NFI equal to 0.851 was also measured, meeting the recommended threshold for NFI [5].

### 2.3 Path model

The PLS estimation path coefficients values as well as the item loadings for the research construct are shown in Fig. 2.

The Microsoft Excel spreadsheet worksheet was used to enter all data and draw conclusions from the data obtained. The Statistical Packages for Social Sciences (SPSS) and the Smart PLS software for structural equation modelling (SEM) technique were used to code data and to run the statistical analysis [6]. Moreover, Smart PLS supports both exploratory and confirmatory research; it is robust to deviations for multivariate normal distributions and is good for a small sample size [6].

## 3 Ethical considerations

This research acted in accordance with the ethical standards of academic research. Hence, an ethical clearance certificate (Ethical clearance number: UFS-HSD2020/0261/1805) was obtained from the University of the Free State General or Human Research Ethics Committee.

## 4 Academic, practical and policy implications of this data article

The present data article offers implications for academicians. The data describes, most notably the relationship between food consumption satisfaction and positive word of mouth. This data is represented by a path coefficient of (β = 0.952), a T-Statistic of 21.966 and a P value of 0.000. This discovery enhances the comprehension of retail marketing in terms of the food consumption. Policy makers and practitioners in the retail space stand to benefit from understanding factors associated with quick service restaurants.

## Declaration of competing Interest

The authors declare that they have no known competing financial interests or personal relationships which have, or could be perceived to have, influenced the work reported in this article.

## Appendix Supplementary materials

Image, application 1

Image, application 2

Image, application 3
