# Supplementary material for: From selected multi-sensory dimensions to positive word of mouth: Data on what really drives generation z consumers to be attached to quick service restaurants in bloemfontein, south africa?
Source: Data Brief. 2020 Sep 7;32:106279. doi: 10.1016/j.dib.2020.106279 (PMC7494664; doi:10.1016/j.dib.2020.106279)
Supplement: Supplementary file 3 [file mmc3.docx]

**ANNEXURE 1: QUESTIONNAIRE**

**University of the Free State**


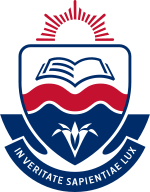


**Participant Information Sheet**

Good day Madam/Sir

I am Dr Eugine Tafadzwa Maziriri a Senior Lecturer within the Department of Business Management, at the University of the Free State. I am conducting a research project titled, “FROM SELECTED MULTI-SENSORY DIMENSIONS TO POSITIVE WORD OF MOUTH: DATA ON WHAT REALLY DRIVES GENERATION Z CONSUMERS TO BE ATTACHED TO QUICK SERVICE RESTAURANTS IN BLOEMFONTEIN, SOUTH AFRICA?” The study will examine how selected multi-sensory dimensions (sight, sound and smell) would influence consumer attitudes towards quick-service restaurants, restaurant patronage intention, food purchase decision, food consumption satisfaction, restaurant attachment, repurchase intention and positive word of mouth.

There will be no wrong or right answers. You are therefore invited to please participate in the study. It will take about 10 to 20 minutes of your time to respond. Please note that your involvement is completely voluntary and your refusal to participate will not result in any penalty. I assure you that the survey data are only for academic research and won’t be used for any commercial purpose. All information collected will be anonymous and responses will be kept confidential. You can refuse to answer any questions that you are not comfortable with and can withdraw at any time you wish to.

Section A of this survey captures some demographic data. Please make a cross (X) in the appropriate box. Section B through D capture the selected multisensory dimensions, while section E through K capture consumer attitudes towards quick-service restaurants, restaurant patronage intention, food purchase decision, food consumption satisfaction, restaurant attachment, repurchase intention and positive word of mouth, respectively. Please indicate the extent to which you agree with each statement by making a cross (X) in the appropriate box.

Thank you for considering participating in this survey. Should you have any questions, or should you wish to obtain a copy of the results of the survey, please contact me on the following details.

Kind regards,

# Dr Eugine T. Maziriri

# Email: maziririet@ufs.ac.za

# Tel: 051-401-7856

# SECTION A

**DEMOGRAPHIC INFORMATION**

This section includes general biographical questions. Please indicate your answer by marking an

(X) on the appropriate box.

# A1. Please indicate your age

| 1 | 18 years old |  |
| --- | --- | --- |
| 2 | 19 years old |  |
| 3 | 20 years old |  |
| 4 | 21 years old |  |
| 5 | 22 years old |  |
| 6 | 23 years old |  |
| 7 | 24 years old |  |
| 8 | 25 years old |  |

**A2. Please indicate your gender**

| 1 | Male |  |
| --- | --- | --- |
| 2 | Female |  |
| 3 | Prefer not to say |  |

**A3. Please indicate your current year of study.**

| 1 | 1st year |  |
| --- | --- | --- |
| 2 | 2nd year |  |
| 3 | 3rd year |  |
| 4 | Post graduate study |  |

**A4. How much allowance do you receive per month?**

| 1 | Less than R500 |  |
| --- | --- | --- |
| 2 | R501 – R1000 |  |
| 3 | R1001-R1500 |  |
| 4 | R1501-R2000 |  |
| 5 | More than R2000 |  |

**A5. How often do you eat from quick-service restaurants? Please indicate below:**

| 1 | Everyday |  |
| --- | --- | --- |
| 2 | A few times a week |  |
| 3 | A few times a month |  |
| 4 | Once in a while |  |

**A6. Which ONE of the following is your most visited/favourite quick-service restaurant? Please indicate below**

| 1 | KFC |  |
| --- | --- | --- |
| 2 | Chicken licken |  |
| 3 | McDonald’s |  |
| 4 | Kara Nichas |  |
| 5 | Sizzler’s |  |
| 6 | Debonairs |  |
| 7 | Steers |  |
| 8 | Other |  |

If other, please specify: _

# A7. Reason for visiting quick-service restaurants

Please cross only **ONE** below

| 1 | I visit quick-service restaurants to eat breakfast/ lunch/dinner |  |
| --- | --- | --- |
| 2 | I socialise in quick-service restaurants |  |
| 3 | I visit quick-service restaurants to get take-out |  |

For all the following sections. Please indicate the extent to which you disagree/agree with the following statements using a cross (X).

# SECTION B: SMELL (SM)

|  |  | Strongly  disagree | Disagree | Neutral | Agree | Strongly  agree |
| --- | --- | --- | --- | --- | --- | --- |
| SM1 | My favourite restaurant has a pleasant scent. | 1 | 2 | 3 | 4 | 5 |
| SM2 | My favourite restaurant has an intense scent (aroma). | 1 | 2 | 3 | 4 | 5 |
| SM3 | My favourite restaurant has a familiar scent. | 1 | 2 | 3 | 4 | 5 |

**SECTION C: SIGHT (ST)**

|  |  | Strongly  disagree | Disagree | Neutral | Agree | Strongly  agree |
| --- | --- | --- | --- | --- | --- | --- |
| ST1 | The inside of the restaurant is bright. | 1 | 2 | 3 | 4 | 5 |
| ST2 | The inside of the restaurant is colourful. | 1 | 2 | 3 | 4 | 5 |
| ST3 | The inside of the restaurant is stimulating. | 1 | 2 | 3 | 4 | 5 |
| ST4 | The inside of the restaurant is lively. | 1 | 2 | 3 | 4 | 5 |
| ST5 | The inside of the restaurant is cheerful. | 1 | 2 | 3 | 4 | 5 |
| ST6 | The inside of the restaurant is interesting. | 1 | 2 | 3 | 4 | 5 |

| ST7 | The inside of the restaurant is comfortable. | 1 | 2 | 3 | 4 | 5 |
| --- | --- | --- | --- | --- | --- | --- |
| ST8 | The inside of the restaurant is relaxed. | 1 | 2 | 3 | 4 | 5 |

**SECTION D: SOUND (SD)**

|  |  | Strongly  disagree | Disagree | Neutral | Agree | Strongly  agree |
| --- | --- | --- | --- | --- | --- | --- |
| SD1 | I often notice the music that plays in the restaurant | 1 | 2 | 3 | 4 | 5 |
| SD2 | The music that plays in restaurant is important to me. | 1 | 2 | 3 | 4 | 5 |
| SD3 | The music inside the restaurant needs to suit my taste. | 1 | 2 | 3 | 4 | 5 |
| SD4 | The music inside the restaurant needs to reflect  the brand’s signature. | 1 | 2 | 3 | 4 | 5 |
| SD5 | Pleasant music creates a favourably atmosphere. | 1 | 2 | 3 | 4 | 5 |
| SD6 | Pleasant music will make  me stay in the restaurant for longer. | 1 | 2 | 3 | 4 | 5 |
| SD7 | Music that is not my taste will make me stay in the restaurant for shorter. | 1 | 2 | 3 | 4 | 5 |
| SD8 | Loud music in the restaurant annoys me. | 1 | 2 | 3 | 4 | 5 |
| SD9 | I like loud music in the restaurant it creates a  pleasant in-store experience. | 1 | 2 | 3 | 4 | 5 |

**SECTION E: CONSUMER ATTITUDES TOWARDS A RESTAURANT (CATR)**

|  |  | Strongly  disagree | Disagree | Neutral | Agree | Strongly  agree |
| --- | --- | --- | --- | --- | --- | --- |
| CATR 1 | The restaurant is up to speed with the food trends | 1 | 2 | 3 | 4 | 5 |

|  | (compared to similar restaurants or other  restaurants in the same category) |  |  |  |  |  |
| --- | --- | --- | --- | --- | --- | --- |
| CATR 2 | The restaurant is a fine-quality establishment (compared to similar restaurants or other restaurant in the same category | 1 | 2 | 3 | 4 | 5 |
| CATR3 | The overall value of dining at the restaurant seems high (compared to similar  restaurants or other restaurants the same category) | 1 | 2 | 3 | 4 | 5 |

**SECTION F: RESTAURANT PATRONAGE INTENTION**

|  |  | Strongly  disagree | Disagree | Neutral | Agree | Strongly  agree |
| --- | --- | --- | --- | --- | --- | --- |
| RPI1 | The likelihood of eating in  the restaurant is high | 1 | 2 | 3 | 4 | 5 |
| RPI2 | The probability that I would  consider eating in the restaurant is high. | 1 | 2 | 3 | 4 | 5 |
| RPI3 | My willingness to eat in the  restaurant is high. | 1 | 2 | 3 | 4 | 5 |

**SECTION G: FOOD PURCHASE DECISION (FPD)**

|  |  | Strongly  disagree | Disagree | Neutral | Agree | Strongly  agree |
| --- | --- | --- | --- | --- | --- | --- |
| FPD1 | I feel good about my decision to purchase food from this restaurant. | 1 | 2 | 3 | 4 | 5 |
| FPD2 | I will positively recommend this restaurant to other people | 1 | 2 | 3 | 4 | 5 |

| FPD3 | I will frequently purchase food from this restaurant | 1 | 2 | 3 | 4 | 5 |
| --- | --- | --- | --- | --- | --- | --- |
| FPD4 | I intent to purchase food again from this restaurant in  the future | 1 | 2 | 3 | 4 | 5 |
| FPD 5 | Overall, I am satisfied about my purchase of food from this restaurant | 1 | 2 | 3 | 4 | 5 |

**SECTION H: FOOD CONSUMPTION STATISFACTION (FCS)**

|  |  | Strongly  disagree | Disagree | Neutral | Agree | Strongly  agree |
| --- | --- | --- | --- | --- | --- | --- |
| FCS1 | I am satisfied with the food  from this restaurant | 1 | 2 | 3 | 4 | 5 |
| FCS2 | I am satisfied with my decision to be consuming  food | 1 | 2 | 3 | 4 | 5 |
| FCS3 | Consuming food from this  restaurant makes me happy | 1 | 2 | 3 | 4 | 5 |
| FCS4 | I am pleased that I am  consumer of this restaurant | 1 | 2 | 3 | 4 | 5 |
| FCS5 | I am relatively satisfied  with my consumption of food from this restaurant | 1 | 2 | 3 | 4 | 5 |

**SECTION I: RESTAURANT ATTACHMENT (RA)**

|  |  | Strongly  disagree | Disagree | Neutral | Agree | Strongly  agree |
| --- | --- | --- | --- | --- | --- | --- |
| RA1 | I feel this restaurant is a part of me. | 1 | 2 | 3 | 4 | 5 |
| RA2 | I identify strongly with this restaurant | 1 | 2 | 3 | 4 | 5 |

| RA3 | Visiting this restaurant says a lot about who I am | 1 | 2 | 3 | 4 | 5 |
| --- | --- | --- | --- | --- | --- | --- |
| RA4 | I am very attached to this restaurant. | 1 | 2 | 3 | 4 | 5 |
| RA5 | I feel a strong sense of belonging  to this restaurant | 1 | 2 | 3 | 4 | 5 |
| RA6 | This restaurant means a lot to me. | 1 | 2 | 3 | 4 | 5 |

**SECTION J: REPURCHASE INTENTION (RI)**

|  |  | Strongly  disagree | Disagree | Neutral | Agree | Strongl  y agree |
| --- | --- | --- | --- | --- | --- | --- |
| RI1 | I am willing to buy food again from this restaurant | 1 | 2 | 3 | 4 | 5 |
| RI2 | I intend to continue purchasing food on a regular basis | 1 | 2 | 3 | 4 | 5 |
| RI3 | I do not plan to stop buying food from this restaurant | 1 | 2 | 3 | 4 | 5 |
| RI4 | It is likely that I will continue purchasing food in the future. | 1 | 2 | 3 | 4 | 5 |

**SECTION K: POSITIVE WORD OF MOUTH (PW)**

|  |  | Strongly  disagree | Disagree | Neutral | Agree | Strongly  agree |
| --- | --- | --- | --- | --- | --- | --- |
| PW1 | I have told others about this restaurant | 1 | 2 | 3 | 4 | 5 |
| PW2 | I would recommend the restaurant to others | 1 | 2 | 3 | 4 | 5 |
| PW3 | I am willing to inform others if they ask me about the restaurant | 1 | 2 | 3 | 4 | 5 |
| PW4 | I will strongly recommend the restaurant to others | 1 | 2 | 3 | 4 | 5 |

*Thank you for your time and patience in completing this survey*
